# GPCR-Based Bioactive Peptide Screening Using Phage-Displayed Peptides and an Insect Cell System for Insecticide Discovery

**DOI:** 10.3390/biom11040583

**Published:** 2021-04-16

**Authors:** Man-Yeon Choi, Robert K. Vander Meer

**Affiliations:** 1 USDA-ARS, Horticultural Crops Research Laboratory, Corvallis, OR 97330, USA; 2 USDA-ARS, Center for Medical, Agricultural, and Veterinary Entomology, Gainesville, FL 32608, USA; bob.vandermeer@usda.gov

**Keywords:** GPCR, neuropeptide, bioactive peptides, phage-displayed peptides, biopanning, insect cell expression, insecticide discovery, RECEPTORi

## Abstract

The discovery of new insecticides improves integrated pest management (IPM), but is usually a long high-risk process with a low probability of success. For over two decades, insect neuropeptides (NPs) and their G-protein coupled receptors (GPCRs) have been considered as biological targets for insect pest control, because they are involved in almost all physiological processes associated with insect life stages. A key roadblock to success has been the question of how large volume chemical libraries can be efficiently screened for active compounds. New genomic and proteomic tools have advanced and facilitated the development of new approaches to insecticide discovery. In this study, we report a novel GPCR-based screening technology that uses millions of short peptides randomly generated by bacteriophages, and a method using an insect Sf9 cell expression system. The fire ant is a good model system, since bioactive peptides have been identified for a specific GPCR. The novel small peptides could interfere with the target GPCR-ligand functions. Therefore, we refer to this new mechanism as “receptor interference” (RECEPTORi). The GPCR-based bioactive peptide screening method offers multiple advantages. Libraries of phage-displayed peptides (~10^9^ peptides) are inexpensive. An insect cell-based screening system rapidly leads to target specific GPCR agonists or antagonists in weeks. Delivery of bioactive peptides to target pests can be flexible, such as topical, ingestion, and plant-incorporated protectants. A variety of GPCR targets are available, thus minimizing the development of potential insecticide resistance. This report provides the first proof-of-concept for the development of novel arthropod pest management strategies using neuropeptides, and GPCRs.

## 1. Introduction

The discovery of new insecticides is a long iterative process with high risk and low chances of success [1]. An efficient screening process using large volume chemical libraries, including natural products, is required. It is anticipated that the discovery of novel biologically-based insecticides will be facilitated with technological advancements, such as ‘-omics’ tools. A variety of insect genomes (https://i5k.nal.usda.gov, accessed on 2 February 2020) and RNA sequences are currently available and offer great potential for a genome-based approach for characterizing target G-protein-coupled receptors (GPCRs). This approach, coupled with a technique to screen chemical libraries, is exciting and holds promise for the identification of small pharmacological compounds for pest control [2,3,4].

Most insect neuropeptides (NPs) activate GPCRs, which are involved in a variety of critical physiological processes at all insect life stages, including fat body homeostasis, feeding, digestion, excretion, circulation, reproduction, metamorphosis, and behavior [5,6,7]. For over two decades, insect NPs and their GPCRs have been proposed as potential targets for next-generation pest management [8,9]. The development of GPCR-based assays is dependent on the expression of the targeted GPCR in a recombinant cell system. Ligand binding strength to the GPCRs can be measured by fluorescence intensities induced by second messengers such as Ca^2+^ or cAMP molecules in the cells.

One major insect neuropeptide family is the PRXamide (NH_2_) peptide family (X = a variable amino acid). The peptide family is well-characterized with a common amino acid sequence, PRXamide, at the C-terminal end, which is conserved across animal groups [10]. Data mining and computational analysis from the *Drosophila* genome identified three GPCRs (CG8784, CG8795, CG9918) for PRXamide peptides that are orthologs to the vertebrate neuromedin U receptor (NmU-R) [11,12]. The hypothesis of ligand-receptor coevolution was supported when GPCRs of *Drosophila* PRXamide peptides were first identified and found to be evolutionarily related to the vertebrate peptide, NmU [13,14]. Since then, many GPCRs and their PRXamide ligands have been identified from other insect groups, and have a variety of biological functions [10], e.g., pheromone biosynthesis by the pheromone biosynthesis activating neuropeptide (PBAN), diapause by the diapause hormone (DH), ecdysis by the ecdysis triggering hormone (ETH), and muscle contraction by the pyrokinin (PK) peptide.

Therefore, the PRXamide peptides/receptors are considered good targets for pest control. For example, genes of the PRXamide peptides and their GPCRs from insect pests were selected as RNA interference (RNAi) targets [15,16,17]. In the present study, we used the fire ant as a model system, because the PBAN/PK peptides and their cognate receptor have been well characterized in the fire ant [18]. A novel GPCR-based biopanning technology was developed using an insect cell system expressing the target GPCR to screen small peptides from phage-displayed peptide libraries, based on their binding to the target GPCR. In our proof-of-concept research, small peptides were isolated and identified that act on the GPCR of the PBAN. The small peptides could interfere with the target GPCR-ligand function. We refer to this new mechanism as “receptor interference” (RECEPTORi). The research reported here illustrates how our innovative methodology can rapidly lead to the development of novel arthropod pest management tools using neuropeptides and their GPCRs. This RECEPTORi technology can be extended to other GPCRs and pests in the future.

## 2. Materials and Methods

### 2.1. Insects, Cloning and Expression of the GPCR, and Binding Assay

The fire ant, *Solenopsis invicta*, samples were from monogyne (single functional queen) colonies collected in the Gainesville, FL USA area by nest excavation or by rearing colonies from newly mated queens. All colonies were maintained as described previously [19].

After total RNA was isolated from fire ant adults and the cDNA was synthesized, all procedures for cloning the fire ant GPCR PRXamide peptide were followed as previously described [18]. The GPCR gene was ligated into a pIB/V5-His TOPO expression vector (Thermo Fisher Scientific, Waltham, MA, USA).

The functional expression of the GPCR in Sf9 cells, and subsequent binding assays were followed as previously described [18,20]. Five micrograms of pIB/GPCR plasmid and 15 µL of Cellfectin II reagent (Thermo Fisher Scientific, Waltham, MA, USA) were diluted into 500 µL of serum free Insectagro Sf9 medium (Corning, Corning, NY, USA) and allowed to incubate for 10 min at room temperature, after which they were mixed together and allowed to incubate for an additional 15–20 min. The pIB/GPCR cellfectin mixture was then added to T-25 cell culture flasks containing Sf9 cells at 50% confluency and incubated overnight at 28 °C. The following morning, the media was removed from each flask and replaced with media containing 20 µg/mL of blasticidin (Corning, Corning, NY, USA). Each transfected cell line was cultured in the presence of 20 µg/mL of blasticidin for approximately three weeks until a blasticidin-resistant cell line was established, then the blasticidin concentration was reduced to 10 µg/mL and the cell lines were frozen until use.

### 2.2. Screening of Small Peptides Using Phage Display and Biopanning

The procedure for the screening of small peptides [21,22] includes 8 Steps.

Step 1: Removal of non-specific phages. A 10 µL Ph.D.-C7C phage library (~2 × 10^8^ phage-displayed peptides (New England Biolabs, Ipswich, MA, USA) was transferred into a 1.5 mL tube, and incubated with Sf9 cells (~2 × 10^6^ non-transfected cells) at room temperature for 30 min. Then, the phages bound to the Sf9 cells were collected by centrifugation, at 10,000× *g* for 10 min at 4 °C. The supernatant containing the unbound phages was saved for the next step.

Step 2: Isolation of phages binding to the GPCR. The phage supernatant from Step 1 was transferred to a 1.5 mL tube containing Sf9 cells (~2 × 10^6^) expressing the PBAN GPCR, and incubated at room temperature for 1 h. Then, specific phage peptides bound to Sf9 cells expressing the GPCR were collected along with the cells by centrifugation, at 10,000× *g* for 10 min at 4 °C.

Step 3: Separation of bound phages from the Sf9 cells expressing the GPCR. The supernatant from Step 2 was decanted to collect the cell pellet containing phages bound to the GPCR expressed by the Sf9 cells. The cell pellet was resuspended in 50 mM glycine-HCl (pH 2.2) and incubated at room temperature for 10 min with shaking to separate the phages from the Sf9 cells. Forty-five microliters of 1 M Tris-HCl (pH 9.0) were added to neutralize the phage solution.

Step 4: Transfection of phages into *E. coli*. The phage solution was added into 20 mL of a preincubated (OD 0.4–0.6 at 600 nm) *E. coli* culture (K12 ER2738 strain, New England Biolabs, Ipswich, MA, USA) that contained 20 μg/mL of tetracycline. Then, the *E. coli* culture was incubated at 37 °C for 4.5 h with vigorous shaking.

Step 5: Amplification of phages. After 4.5 h of incubation, the cell culture was centrifuged at 10,000× *g* for 10 min at 4 °C, then the supernatant was collected and transferred to a fresh tube.

Step 6: Harvesting phages. A solution of polyethylene glycol 800 (20%) (Sigma-Aldrich, St. Louis, MO, USA) containing 2.5 M NaCl was added to the supernatant of Step 5, mixed, and then incubated on ice for 2 h.

Step 7: Isolation of phages. The solution from Step 6 was centrifuged at 10,000× *g* for 20 min at 4 °C, and the collected pellet was resuspended in a standard phosphate buffered saline (PBS) with 15% glycerol and was used for the next round of screening.

Steps 1 to 7 were repeated 3–4 times using the isolated phages from Step 7.

**Figure 1:**
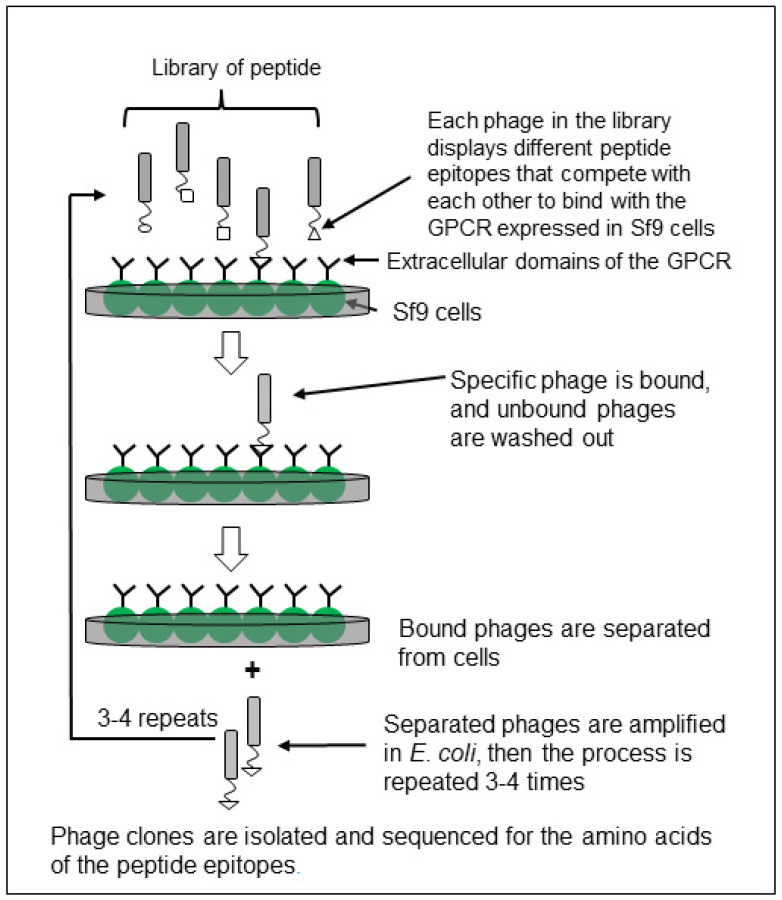
A schematic diagram of GPCR (G-Protein-Coupled Receptor) based screening using a phage display peptide library and biopanning using the insect Sf9 cell expression system. This process requires less than two weeks to obtain amino acid sequences of small peptide molecules after the establishment of functional GPCR expression in the Sf9 insect cell line (see detail in Materials and Methods).

Step 8: Selection of phage colonies. After the 4th round, 20 or more phage colonies were selected and cultured in *E. coli* to amplify the phages (Figure 1). The purified phage DNAs from the culture were sequenced (Interdisciplinary Center for Biotechnology Research, ICBR, University of Florida, Gainesville, FL, USA) to identify nucleotide sequences encoding the peptides.

### 2.3. Preparation of Phage and Ligand Peptides

All peptides used in this study were synthesized with more than 95% purity, to contain an amide group on their C-termini (Peptide 2.0 Inc., Chantilly, VA, USA). Peptides were solubilized in water, aliquoted into 1 to 20 nmol working stocks, dried using a DNA120 SpeedVac Concentrator (Thermo Scientific, Waltham, MA, USA), and frozen at −20 °C until use in various assays.

### 2.4. Bioassays

Injection: A selected peptide was injected into the hemocoel of adult ants using a Nanoliter 2000TM System with pulled borosilicate needles (World Precision Instruments, Sarasota, FL, USA). Synthetic peptides (5 nmol/0.1 µL) were dissolved in a hymenopteran saline (130 mM NaCl, 6 mM KCl, 4 mM MgCl_2_, 5 mM CaCl_2_, 160 mM sucrose, 25 mM glucose, and 10 mM HEPES, pH 7.2). The saline was used only for the control. Ants were observed for mortality for 10–16 days post-injection.

Feeding: Each evaluated peptide was dissolved in a 10% sucrose solution (1 mg/mL). Worker ants (250) were randomly selected from a laboratory reared fire ant. The ants were confined in a small plastic cup of which the upper-inside surface was coated with Fluon to prevent them from escaping. Water was provided via a moistened cotton ball throughout the experiment. The ants in the cups were starved for 24 h prior to introduction of the peptide treatment. Ant bioassay units fed only 10% sucrose solution served as controls. Ants were observed for mortality for 12 days.

### 2.5. Statistical Analysis

Statistical procedures, e.g., log-rank test survival, and graphical representations were carried out using GraphPad Prism, version 6 (GraphPad Software Inc., San Diego, CA, USA). In some situations, statistical significance was determined by inspection.

## 3. Results

### 3.1. Phage Display Library and Biopanning Using Sf9 Cell Expression

Phages bound to the GPCR were then separated, amplified into the host *E. coli*, and recycled through the process another three times. After the fourth round of the panning selection process, the phage DNAs from randomly chosen clones were sequenced to identify the small peptide epitopes. After the target GPCR was expressed in Sf9 insect cells, the GPCR-based screening process required less than two weeks to isolate and identify small peptides (Figure 1) [21,22].

### 3.2. Analysis of Phage-Displayed PEPTIDES Resulting from the Biopanning

Among 58 phage clones sequenced, the most abundant heptapeptide was identified with the epitope ‘QKIGSHF’ (19%, 11 of 58 clones), followed by ‘LKIGSHF’ (12%, 7 of 58 clones), then the three epitopes, ‘IQQGSHF’, ‘ERVGSHF’, and ‘VKLGSHF’ (each at 5%, 3 of 58 clones) (Table 1). Surprisingly, the homology of the peptides gradually changed in their motifs, -KIGSHF, --IGSHF, and ---GSHF at the C-terminal end. From all the peptide epitopes, 30 had the tetrapeptide, ‘GSHF’ (52%). This number increased to 37 epitopes (64%) if serine (S) and threonine (T) are interchanged, GSHF and GTHF (Table 1). The amino acids, S and T, belong to the same group, having a polar and uncharged functional group. The most abundant dipeptide sequence was ‘HF’ at the C-terminal end, found in 40 heptapeptides (69%). Among the 58 heptapeptides, 10 peptide sequences were found at least twice and of these, eight had ‘HF’ at the C-terminal end. Two peptides, MARYMSA and RGATPMS, had unique sequences compared to the others. None of the selected sequences had any similarity to the peptide sequence of the natural fire ant PBAN ligand, GSGEDLSYGDAYEVDEDDHPLFVPRLamide.

### 3.3. Phenotypic Impact of the Novel Peptides on Fire Ants

**Figure 2:**
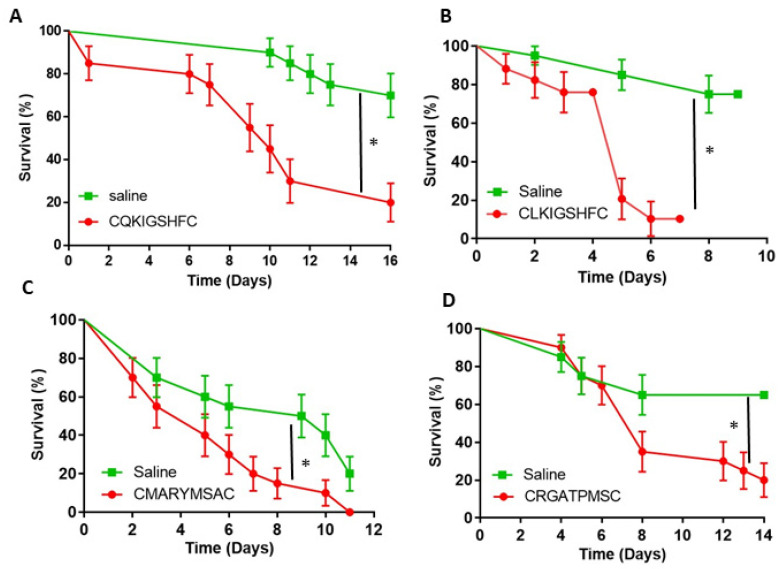
The survival of fire ant workers after injection of synthetic peptides. Saline was injected as a control. An asterisk (*) indicates significant differences by Logrank test (**A**) CQKIGSHFC (*p* = 0.0001), (**B**) CLKIGSHFC (*p* = 0.0001); (**C**) CMARYMSAC (*p* = 0.0066); (**D**) CRGATPMSC (*p* = 0.0180). Survival curves were compared using a standard Log-rank test survival analysis. Each replicate was composed of twenty ants, each treatment/control was replicated three times (mean ± 3 s.d).

The 10 heptapeptides that had at least two identical clones (Table 1) were selected and synthesized with a pair of flanking cysteine residues, thus forming a loop. The first bioassay introduced the peptides into the target fire ant workers via injection, after which the fire ants were observed for phenotypic effects. While injection is not practical as a control method, it is the surest way to get the peptides into the hemolymph of the target ants. Fire ant survival was followed for up to 16 days (Figure 2A–D). Four injected peptides, CQKIGSHFC, CLKIGSHFC, CMARYMSAC, and CRGATPMSC, showed significant impact on fire ant survival compared to the saline control (Log-rank Survival, *p* < 0.05). However, the other six peptides did not have a significant effect (data not shown).

**Figure 3:**
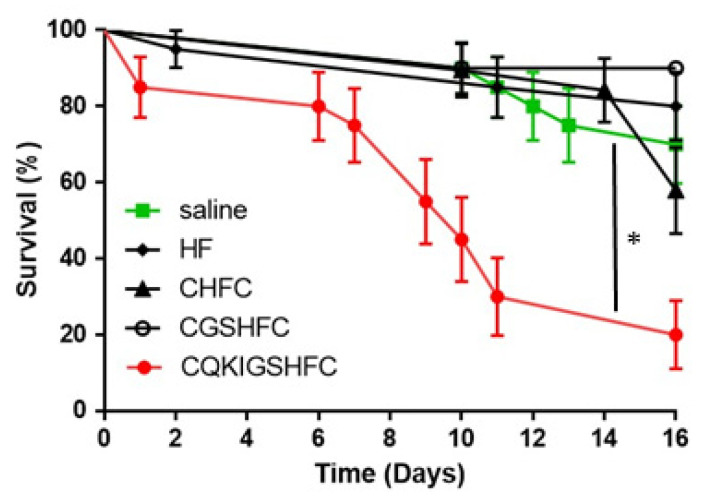
The survival of fire ant workers after injection of the most abundant peptide (CQKIGSHFC) and various short peptides modified from the original epitope. Saline was injected as a control. The asterisk (*) indicates significant differences by Logrank test (CQKIGSHFC (*p* = 0.0001). Survival curves were compared using a standard Log-rank test survival analysis. Each replicate was composed of twenty ants, each treatment/control was replicated three times (mean ± 3 s.d).

Two short modifications of the active epitope, CQKIGSHFC, which is the most abundant peptide resulting from the screening process (Table 1 and Figure 2A), were synthesized with flanking cysteine residues (CGSHFC and CHFC) as an initial step toward the investigation of structure-activity relationships. These two short peptides did not elicit significant ant mortality after injection (Figure 3). In addition, the HF peptide without cysteine flanking did not negatively affect ant survival after injection compared to the control (Figure 3). HF is the most abundant C-terminal dipeptide end sequence found in the peptides isolated from the screening process (Table 1).

**Figure 4:**
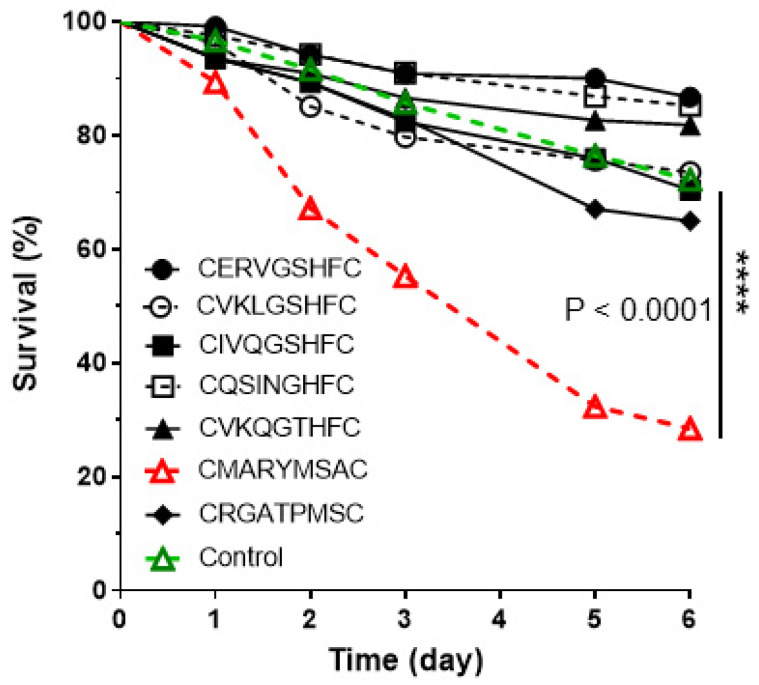
The survival curves of fire ant workers after being fed synthetic peptides. The control was 10% sucrose solution. Each treatment and the control consisted of 250 workers ants. The peptide, CMARYMSAC, showed ~26% survival after 5 days, and indicates significant differences by a standard Log-rank test. The other peptides showed survival rates that were not different from the saline control. After six days, there was no further ant mortality (****: *p* < 0.0001).

Results of the feeding assay with seven peptides showed different survival rates compared to the injection (Figure 4). Only one of seven peptides evaluated, CMARYMSAC, significantly reduced the ant survival rate, ~26% survival within 6 days. After 6 days, there was no further ant mortality. The other six peptides were not different from the saline control (Figure 4).

## 4. Discussion

### 4.1. GPCR-Based Screening Using a Library of Phage-Displayed Peptides and Biopanning

The bacteriophage display library coupled with an insect cell expressing the PBAN GPCR successfully led to the isolation of seven amino acid peptides [23,24,25]. The peptides in the phage displays are randomly generated to a specific number of amino acids, in our case seven amino acids (CXXXXXXXC where X is any amino acid and C is cysteine), creating a vast library (~2 × 10^8^) of peptide sequences, each of which are exposed on their unique phage surface. The phage display technology has been widely used to isolate proteins, antibodies, cell surfaces, agonists, antagonists, and intracellular proteins in protein-protein interactions [26,27,28]. The biopanning method uses a differential centrifugation process to separate interactive ligands for drug discovery and gene delivery agents [29,30,31].

GPCRs are seven transmembrane (7 TM) domain receptors, and are the largest class of cell surface receptors for many key targets in drug development. GPCRs have a structural advantage with the extra cellular binding domains that provide binding pockets for small molecules [32]. The test molecules can be obtained from combinatorial libraries, complex biological mixtures, and/or phage libraries. The common high-throughput screening method for GPCR-based drug discovery uses a variety of chemical libraries, including natural products. Bioactive chemical libraries are commercially available, but they are very expensive (e.g., $20,000 per 100 µL in 10 mM solution). Therefore, use of the phage libraries (e.g., $300 for ~2 × 10^10^/phage library) and GPCR-based biopanning are more feasible and cost-effective models for novel insecticide discovery.

### 4.2. Small Peptides and PRX Peptides

We expected the strong PBAN binding peptides identified from the phage peptide library to contain the PRX motif. Interestingly, none of the amino acid sequences in the heptapeptides are found in the natural ligand for the PBAN receptor, which for the fire ant, is GSGEDLSYGDAYEVDEDDHPLFVPRL [18,33]. Even the conserved C-terminal -PRX or similar epitope was not found among the 58 peptide epitopes identified. All insect PRX family peptides reported to date have the conserved PRXamide in the C-terminal end [10,34]. Therefore, the heptapeptides selected by the methodology in this study may be acting as antagonistic ligands to the GPCR, rather than as agonistic molecules. To evaluate this possibility, we measured the binding activity of each identified peptide to the PBAN GPCR expressed in the Sf9 cells. None of these peptides activated extracellular Ca^2+^ release into the cells, as would be expected for the natural PBAN ligand (data not shown). This result indicates that the isolated phage peptides and the natural ligand are binding to different sites of the GPCR. The phage peptides identified are binding in such a way to either irreversibly change the conformation of the receptor, so the natural ligand cannot bind to the receptor, or the phage peptides are binding with the GPCR and blocking access by the natural ligand. Additional evaluation of antagonistic interactions on the GPCR remains for future research.

The differences in results observed after injection and feeding could be caused by different physiological interactions and the method of delivery to the target receptor. One key factor may be the hydrophobicity of the amino acids in the peptides. In fact, the peptide CMARYMSAC is more hydrophobic than the other peptides and had low water solubility. This characteristic could help this peptide pass from the midgut to the hemolymph, where it can then act on the target receptor. Formulation with lipid-based nanoparticles or modification of the small peptides could increase their impact on ant survival. Degradation of the peptides by midgut enzymes in the ants may also contribute to the feeding results.

### 4.3. GPCR-Based Insecticide Discovery: A Proof of Concept and Feasibility

Most arthropod neuropeptides and their GPCRs have been well conserved during the evolutionary process, and similar molecules are found in vertebrates. Since the first insect GPCR, the diuretic hormone receptor, was discovered from *Manduca sexta* [35], a variety of GPCRs have been identified from insect genome sequences based on the presence of the well conserved 7 TM regions in GPCRs [11,36,37]. In the *Drosophila* genome, ~200 genes (less than 2% of the total genes) encode for GPCRs, including receptors for neurotransmitters, hormones, olfaction, and taste. Approximately 100 genes have been classified as neurotransmitter and/or hormone GPCRs [36]. GPCRs for neuropeptides (NPs) in the class A family are estimated at only 44 genes based on the fly base [38]. However, the number of functional GPCRs actively translated for insect NPs might be much lower than 44 genes, because many NP GPCRs are known to have multiple variants. The variants are translated, but are not expressed or only marginally expressed. For example, a variety of PRX GPCRs were found with 2–3 variants including silencing receptors [39,40,41,42,43]. Therefore, the total GPCRs for all NPs of target insect pests is a manageable size for GPCR-based insecticide discovery.

Another advantage to NPs as biological targets is that insect NPs have multiple biological functions during insect life stages. One of the best examples is the PRXamide family peptides. The PRXamide peptides have a broad range of physiological functions: (1) stimulation of sex pheromone biosynthesis in female moths [44]; (2) induction of melanization in moth larvae [45]; (3) induction of embryonic diapause in the silkworm [46]; (4) termination of the development of pupal diapause in heliothine moths [47], (5) stimulation of visceral muscle contraction in cockroaches [48]; (6) acceleration of puparium formation in the flesh fly [49], and many unknown functions in other insect groups.

## 5. Conclusions

GPCR-based screening technology for the isolation and identification of bioactive peptides using an insect cell expression system has multiple advantages for the discovery of new insecticides compared to RNAi (Table 2). First, the bioactive peptides act to interfere with the normal binding function of the natural GPCR ligand. We call this mechanism ‘receptor interference’ (RECEPTORi). It results in negative effect(s) on insect survival. Secondly, potential NP GPCR candidates in total are estimated to include only 20–30 genes, which would promote the rapid screening of all NP GPCRs in the target pest. Thirdly, screening for bioactive peptides is a rapid process. Once the functional expression of the GPCR is established in an insect cell line, it takes less than two weeks to obtain the amino acid sequences of small peptides. Fourthly, delivery of bioactive peptides to target pests can be flexible, e.g., topical, ingestion, or structural modification such as a cyclic form with flanking cysteines. They can also be formulated for specific applications, e.g., as plant-incorporated protectants, similar to the *Bacillus thuringiensis* (*Bt*) toxin. The ease of using the methodology described here opens up the possibility of developing antagonists for multiple key GPCR functions within a single insect species.

## 6. Patents

There are two patents (U.S. Patent No: 9,771,393 B2 and U.S. Patent No: 10,017,358 B2) resulting from the work reported in this manuscript.

## Figures and Tables

**Table 1:** The peptides screened from the phage display libraries after 3–4 selection rounds are shown. The library heptapeptides are loop-constrained by a pair of flanking cysteine (C) residues. The most abundant amino acids, G, S, H and F, are in green print.

Phage Sequence (CXXXXXXXC)	Abundance of Phage Clones	Phage Sequence (CXXXXXXXC)	Abundance of Phage Clones
**QKIGSHF**	11	**ERVGTHY**	1
**LKIGSHF**	7	**IKVGPHY**	1
**IQQGSHF**	3	**IKIGSHY**	1
**ERVGSHF**	3	**QRIGLHY**	1
**VKLGSHF**	2	**VSRTSHL**	1
**IVQGSHF**	2	**LPWQIHN**	1
**TXVGSHF**	1	**LPMTKHV**	1
**IHIGSHF**	1	**TNANHYF**	1
**VKQGTHF**	2	**QQTKNYY**	1
**TQIGTHF**	1	**SQLPWYS**	1
**IQIGTHF**	1	**MARYMSA**	2
**QSIGTHF**	1	**RGATPMS**	2
**ERVGTHF**	1	**NTGGSMA**	1
**IHXGTHF**	1	**NTGSPYE**	1
**QSINGHF**	2	**HSRVSGT**	1
**YSSPSHF**	1	**TNGDSAR**	1

**Table 2:** Comparison of RECEPTORi and RNAi.

Characteristics	RECEPTORi	RNAi
Target site	GPCR drive	mRNA drive
Mode of action	Interfere receptor	Interfere mRNA
Potential target genes Feasible target genes Average molecular weight	~400–600 genes ~20–30 genes ~1–8 amino acids (MW < 900)	~20,000–40,000 genes ~200–400 genes ~21–500 nucleotides (MW > 13K)
Screening period for targets	Rapid	Slow
Delivery method		
- Topical	Available (after formulation)	Limited due to molecule size
- transgenic (plant-incorporated)	Available	Available
- Ingestion (oral)	Available	Available
- Spraying plant	Available	Available
*Bt* alternative	Possible	Limited (synergistic)
Synthetic mimic (chemical)	Possible	Limited
Target insect	Unlimited	Unlimited
Biodegradable Resistance development	Possible (rapid) Slow	Possible Moderate

## Data Availability

No new data were created or analyzed in this study. Data sharing is not applicable to this article.
